# Environmental endocrine disruptors in cardio-oncology: emerging modifiers of cardiovascular vulnerability in patients with cancer

**DOI:** 10.1186/s40959-026-00532-9

**Published:** 2026-06-19

**Authors:** Vincenzo Quagliariello, Massimiliano Berretta, Giuseppe D’Aiuto, Irma Bisceglia, Matteo Barbato, Benedetta Del Toro, Jacopo Santagata, Maria Laura Canale, Andrea Paccone, Alessandro Inno, Cristiana D’Ambrosio, Stefano Oliva, Christian Cadeddu Dessalvi, Tiziana Di Matola, Domenico Gabrielli, Iacopo Fabiani, Leopoldo Pagliani, Cezar A. Iliescu, Anna Borowiec, Nicola Maurea

**Affiliations:** 1 Division of Cardiology, Istituto Nazionale Tumori- IRCCS- Fondazione G. Pascale, Naples, Italy; 2 Department of Clinical and Experimental Medicine, University of Messina, Messina, Italy; 3 Breast Unit, Clinica Villa Fiorita, Aversa, Caserta Italy; 4 Servizi Cardiologici Integrati, Dipartimento Cardio-Toraco-Vascolare, Azienda Ospedaliera San Camillo Forlanini, Rome, 00152 Italy; 5 U.O.C. Cardiologia, Ospedale Versilia, Lido di Camaiore, Italy; 6 Department of Oncology, Sacro Cuore Don Calabria Hospital (IRCCS), Negrar, 37024 Italy; 7 Cardiology Division, “F. Veneziale”, Molise Regional Health Company (ASREM), Isernia, 86170 Italy; 8 Cardio-Oncology Unit, IRCCS Istituto Tumori, “Giovanni Paolo II”, Bari, 70124 Italy; 9 Department of Medical Sciences and Public Health, University of Cagliari, Cagliari, 09124 Italy; 10 U.O.C. Biochimica Clinica, AORN Ospedali Dei Colli-Monaldi-Cotugno-CTO, Naples, 80131 Italy; 11 U.O.C. Cardiologia, Dipartimento Cardio-Toraco-Vascolare, Azienda Ospedaliera San Camillo Forlanini, Roma-Fondazione Per Il Tuo Cuore-Heart Care Foundation, Florence, 50121 Italy; 12 Health Science Interdisciplinary Research Center, Scuola Superiore Sant’Anna, Pisa, 56127 Italy; 13 Veneto Institute of Oncology I.O.V. - IRCCS, Padua, Italy; 14 Department of Cardiology, The University of Texas MD Anderson Cancer Center, Houston, US; 15 Maria Sklodowska-Curie National Research Institute of Oncology, Warsaw, Poland

## Abstract

**Background:**

Cardio-oncology has traditionally focused on treatment-related cardiovascular toxicity and conventional cardiovascular risk factors. However, increasing evidence suggests that environmental exposures may contribute to both cancer development and cardiovascular disease through shared biological mechanisms. Endocrine-disrupting chemicals (EDCs) are ubiquitous environmental contaminants capable of interfering with hormonal signaling, metabolic homeostasis, vascular function, and inflammatory pathways. Despite growing evidence linking EDCs to cardiometabolic disorders and hormone-sensitive malignancies, their potential role within the cardio-oncology continuum remains largely unexplored.

**Methods:**

This narrative review summarizes and critically discusses current experimental, translational, and epidemiological evidence regarding the potential contribution of environmental endocrine disruptors to cardiovascular risk and cancer biology. Particular attention is given to molecular pathways relevant to cancer therapy-related cardiovascular toxicity, breast cancer biology, adipose tissue dysfunction, and emerging exposomic determinants of long-term cardiovascular outcomes in patients with cancer.

**Main body:**

Major classes of EDCs, including bisphenols, phthalates, per- and polyfluoroalkyl substances (PFAS), persistent organic pollutants (POPs), parabens, pesticides, and other environmental contaminants, are continuously encountered through food systems, plastics, consumer products, contaminated water, and healthcare materials. These compounds influence multiple biological processes that are central to both oncogenesis and cardiovascular disease, including oxidative stress, mitochondrial dysfunction, endothelial injury, chronic inflammation, metabolic reprogramming, thrombosis, and epigenetic remodeling. In breast cancer, EDCs may modulate subtype-specific signaling pathways involving estrogen receptor activation, HER2 crosstalk, aryl hydrocarbon receptor signaling, and homologous recombination networks. In parallel, growing evidence supports associations between EDC exposure and hypertension, accelerated atherosclerosis, heart failure, metabolic syndrome, and major adverse cardiovascular events. We further discuss the hypothesis that lipophilic EDCs may accumulate within adipose depots and that dysfunctional epicardial adipose tissue could represent a local toxicological niche capable of amplifying cardiovascular vulnerability in cancer survivors, although direct evidence remains unavailable.

**Conclusions:**

EDCs should not yet be considered established cardio-oncology risk factors; however, they represent biologically plausible exposomic modifiers operating at the intersection of cancer, metabolism, and cardiovascular disease. Incorporating environmental exposures into cardio-oncology research may improve understanding of interindividual variability in cardiovascular outcomes and open new avenues for risk stratification, prevention, and survivorship care. Future prospective studies integrating exposure biomarkers, adipose tissue biology, and cardiovascular phenotyping are warranted to define the clinical relevance of EDCs in patients with cancer.

## Introduction

Over the past decades, advances in early detection, targeted therapies, and supportive care have led to a substantial and sustained increase in the number of cancer survivors worldwide [1]. At the same time, a concerning epidemiologic shift has emerged, with a rising incidence of cancer in younger populations [2]. These parallel trends have reshaped the clinical landscape of oncology: cancer is increasingly becoming a chronic condition, and long-term survivorship is now accompanied by a growing burden of non-cancer comorbidities. Among these, cardiovascular disease (CVD) has emerged as a leading cause of morbidity and mortality, often rivaling or exceeding the risk of cancer recurrence in selected populations [3]. This evolving scenario has positioned cardio-oncology as a critical discipline aimed at understanding, preventing, and managing the cardiovascular consequences of cancer and its treatment. Traditionally, cardiovascular risk in oncology has been attributed primarily to the direct and indirect effects of anticancer therapies, including anthracyclines, HER2-targeted agents, radiotherapy, and more recently, targeted therapies and immunotherapies [4]. These treatments can induce a broad spectrum of cardio-vascular toxicities, ranging from myocardial dysfunction and heart failure to hypertension, thrombosis, arrhythmias, and accelerated atherosclerosis [5]. However, this therapy-centric model is increasingly recognized as incomplete. There is growing awareness that baseline patient characteristics, including age, metabolic status, obesity, and pre-existing vascular disease, interact with treatment-related stressors to determine cardiovascular outcomes. Therefore, a novel and still underexplored dimension is the role of the environmental exposome, and in particular, endocrine disruptors (EDs), as potential modifiers of both oncologic and cardiovascular risk [6]. EDs are exogenous chemicals capable of interfering with hormonal signaling and metabolic regulation, and they are now widely distributed in modern environments, including food systems, water, consumer products, and healthcare materials. Importantly, human exposure is not episodic but continuous, occurring through complex mixtures of compounds across the lifespan [7]. Emerging data suggest that such exposures may contribute not only to carcinogenesis, particularly in hormone-sensitive cancers, but also to cardiometabolic dysfunction, endothelial injury, inflammation, and vascular disease [8]. A particularly intriguing hypothesis is that the increasing burden of cancer in younger individuals, as well as the long-term health trajectory of cancer survivors, may be influenced, at least in part, by chronic environmental exposures, including EDs [9, 10]. While this relationship is multifactorial and not yet fully defined, EDs have been implicated in obesity, insulin resistance, dyslipidemia, and chronic inflammation, all of which are recognized risk factors for both cancer development and cardiovascular disease [11]. In this sense, EDs may represent a shared upstream determinant linking oncogenesis and cardiovascular vulnerability. Within cardio-oncology, this raises a critical and largely unaddressed question: could EDs act as silent co-determinants of treatment-related cardiovascular toxicity and long-term outcomes? Mechanistically, EDs converge on many of the same biological pathways implicated in cardiotoxicity, including oxidative stress, mitochondrial dysfunction, endothelial impairment, inflammatory signaling, and epigenetic remodeling [12]. These overlapping pathways suggest that chronic ED exposure may lower the threshold for cardiovascular injury in patients undergoing cancer therapy, potentially amplifying the effects of established cardiotoxic agents [13].

Furthermore, the interplay between EDs and host metabolic state may be particularly relevant. Many EDs are lipophilic and may accumulate in adipose tissue, which itself is an active endocrine organ involved in systemic inflammation and metabolic regulation. In patients with obesity or metabolic syndrome, conditions highly prevalent among cancer survivors, this may create a biologically active reservoir capable of modulating both tumor behavior and cardiovascular risk over time [14]. The potential role of epicardial adipose tissue as a local mediator of these effects adds an additional layer of complexity, particularly in relation to myocardial and coronary vulnerability [15]. Despite these compelling mechanistic and epidemiologic signals, the integration of EDs into cardio-oncology remains in its infancy [16]. The current literature is fragmented, with robust data available separately on EDs and cardiovascular disease, and on EDs and cancer biology, but very limited work explicitly bridging these domains. This gap represents both a challenge and an opportunity. In this review, we aim to provide a comprehensive and hypothesis-driven synthesis of the role of environmental endocrine disruptors in cardio-oncology. We will first examine the chemistry and sources of EDs, emphasizing real-world exposure patterns. We will then explore their biological effects in cardiovascular and cancer-related pathways, with particular attention to mechanisms relevant to cardiotoxicity. Finally, we will discuss emerging clinical im-plications, including potential strategies for exposure reduction and their integration into preventive cardio-oncology.

## Environmental sources of endocrine disruptors: daily exposure in the cardio-oncology era

Notably, EDCs are ubiquitous environmental contaminants capable of interfering with hormonal signaling, metabolic regulation, vascular biology, and cellular homeostasis [17]. Importantly, human exposure does not occur through isolated compounds but rather through chronic, lifelong contact with complex mixtures of endocrine-active chemicals [18]. This concept, now encompassed within the broader framework of the environmental exposome, is particularly relevant to cardio-oncology, where long-term cardiovascular outcomes are increasingly recognized as the result of interactions between cancer therapies, host susceptibility, lifestyle factors, and environmental exposures [19]. Recent estimates suggest that humans are exposed daily to hundreds of synthetic chemicals with known or suspected endocrine-disrupting properties. Although only a fraction have been extensively characterized, the most relevant classes include bisphenols, phthalates, per- and polyfluoroalkyl substances (PFAS), persistent organic pollutants (POPs), parabens, alkylphenols, brominated flame retardants, organophosphate flame retardants, pesticides, ultraviolet filters, and selected metals with endocrine activity [20, 21]. These compounds differ substantially in chemical structure and toxicokinetics but often converge on common biological pathways involving oxidative stress, mitochondrial dysfunction, inflammation, endothelial injury, metabolic reprogramming, and epigenetic remodeling (Table 1).

**Table 1:** The human endocrine disruptor exposome: major chemical classes, everyday exposure sources, and potential relevance to cardio-oncology. Human exposure occurs through complex mixtures of persistent and non-persistent chemicals derived from food systems, water, consumer products, indoor environments, occupational settings, and healthcare-related materials. Several EDC classes converge on biological pathways implicated in both cancer progression and cardiovascular disease, including oxidative stress, endothelial dysfunction, inflammation, metabolic dysregulation, thrombosis, and epigenetic remodeling

Class of EDCs	Representative Compounds	Main Sources of Exposure	Potential Cardio-Oncology Relevance
Bisphenols (Phenolic compounds)	BPA, BPS, BPF, BPAF	Food can linings, polycarbonate plastics, reusable bottles, food storage containers, thermal paper receipts, epoxy resins, coffee machine components	Estrogen receptor activation, endothelial dysfunction, oxidative stress, metabolic dysregulation, potential amplification of therapy-related cardiovascular injury
Phthalates	DEHP, DBP, BBP, DINP, DIDP	Food packaging, cling films, plastic wraps, food-processing equipment, cosmetics, fragrances, flooring, IV tubing, medical devices	Anti-androgenic activity, insulin resistance, hypertension, vascular inflammation, adipose dysfunction, potential cardiotoxic synergy
PFAS (Per- and Polyfluoroalkyl Substances)	PFOA, PFOS, PFHxS, PFNA, GenX	Contaminated drinking water, non-stick cookware, grease-resistant food packaging, stain-resistant textiles, firefighting foams	Persistent bioaccumulation, dyslipidemia, endothelial dysfunction, inflammation, atherosclerosis, increased cardiovascular risk
Persistent Organic Pollutants (POPs)	PCBs, Dioxins, Furans, DDT/DDE, Hexachlorobenzene (HCB)	Contaminated food (especially animal fats), soil, industrial pollution, sediments, water	Lipophilic accumulation in adipose tissue, chronic inflammation, mitochondrial dysfunction, cardiometabolic and oncogenic effects
Parabens	Methylparaben, Ethylparaben, Propylparaben, Butylparaben	Cosmetics, lotions, shampoos, pharmaceuticals, personal-care products	Weak estrogenic activity, chronic dermal exposure, potential relevance in hormone-sensitive cancers
Alkylphenols	Nonylphenol, Octylphenol	Industrial detergents, plastics, food packaging, textile processing	Estrogenic activity, endocrine disruption, vascular inflammation
Benzophenones (UV Filters)	Oxybenzone (BP-3), Benzophenone-1, Benzophenone-3	Sunscreens, cosmetics, personal-care products, plastics, food packaging inks	Estrogenic and anti-androgenic activity, potential relevance in breast cancer biology
Triclosan and Triclocarban	Triclosan, Triclocarban	Antibacterial soaps, toothpaste, cosmetics, household products	Thyroid disruption, microbiome alterations, immune dysregulation
Flame Retardants (Brominated and Organophosphate)	PBDEs, TDCIPP, TPHP	Furniture, electronics, mattresses, carpets, indoor dust	Neuroendocrine disruption, adipose dysfunction, oxidative stress, chronic indoor exposure
Pesticides and Herbicides	Atrazine, Chlorpyrifos, Pyrethroids	Agricultural exposure, food residues, contaminated water, occupational settings	Hormonal interference, oxidative stress, inflammation, potential carcinogenic effects
Heavy Metals and Metalloids with Endocrine Activity	Cadmium, Arsenic, Lead, Mercury	Contaminated water, food, tobacco smoke, industrial emissions, air pollution	Endocrine mimicry, oxidative stress, endothelial dysfunction, atherosclerosis and carcinogenesis
Perchlorate	Perchlorate salts	Drinking water, dairy products, leafy vegetables	Thyroid hormone disruption through inhibition of iodide uptake
Organotins (Obesogens)	Tributyltin (TBT), Dibutyltin	Marine paints, plastics, contaminated seafood	PPARγ activation, adipogenesis, metabolic syndrome, adipose tissue dysfunction
Synthetic Musks	Galaxolide, Tonalide	Perfumes, detergents, cosmetics, household products	Bioaccumulative endocrine-active compounds with potential mitochondrial effects
Microplastic-Associated Chemicals	Phthalates, Bisphenols, Stabilizers, Plastic Additives	Food, drinking water, seafood, airborne particles, indoor dust	Carrier of multiple EDCs, inflammation, oxidative stress, endothelial injury
Medical Device-Related Plasticizers	DEHP, DINCH, Alternative Plasticizers	Intravenous bags, infusion tubing, catheters, blood bags, enteral nutrition systems	Unique exposure source in oncology patients undergoing repeated infusions, hospitalization, and device-intensive care

### Food systems and food packaging: the major source of daily exposure

For most individuals, food represents the principal route of EDC exposure [22]. Contemporary food systems introduce endocrine-active chemicals at multiple stages, including production, processing, storage, packaging, transportation, and preparation [23]. Bisphenols remain among the most extensively studied contaminants and may migrate from epoxy resin coatings used in food cans, beverage containers, reusable polycarbonate bottles, coffee machine components, and thermal paper receipts. Importantly, increasing evidence indicates that BPA substitutes such as bisphenol S (BPS) and bisphenol F (BPF) may retain similar endocrine activity despite being marketed as safer alternatives [23, 24]. Phthalates are particularly relevant because they are not chemically bound to plastic matrices and readily leach into food [25]. Exposure may occur through food-processing equipment, conveyor belts, gloves used during food preparation, plastic tubing, takeaway containers, cling films, and vacuum-packed products. Fat-rich foods, including cheese, processed meats, fast food, dairy products, and oils, frequently exhibit higher concentrations because phthalates are highly lipophilic (Table 1). Less widely appreciated sources include:

- Tea bags containing plastic polymers;
- Coffee capsules and single-use coffee pods;
- Microwave popcorn bags;
- Grease-resistant bakery wrappers;
- Pizza boxes coated with fluorinated compounds;
- Paper straws treated with PFAS-containing coatings;
- Reusable plastic sports bottles repeatedly exposed to heat.

These examples illustrate how exposure may occur even among individuals attempting to follow healthy dietary habits [26].

### Water, beverages, and hidden chemical contamination

Water is increasingly recognized as a major vehicle for EDC exposure [27]. PFAS contamination has emerged as a global public health concern, particularly near industrial facilities, airports, military bases, landfills, and firefighting training sites [28]. Due to their exceptional environmental persistence, PFAS have been detected in groundwater, drinking water supplies, rivers, seafood, and agricultural products worldwide [29]. Less commonly recognized sources include:

- Domestic water filtration systems containing fluorinated components;
- Bottled water stored under high temperatures;
- Ice produced from contaminated municipal water;
- Beverages dispensed through polymer-containing tubing systems;
- Sports and energy drinks stored for prolonged periods in plastic packaging.

Perchlorate contamination of groundwater represents an additional concern because of its capacity to interfere with thyroid hormone synthesis through inhibition of iodide uptake [30] (Table 1).

### Indoor environments: the forgotten exposure route

Peoples in industrialized countries spend approximately 90% of their time indoors, making indoor environments an important yet frequently overlooked source of endocrine disruptor exposure [31]. Household dust contains a complex mixture of phthalates, PFAS, brominated flame retardants, organophosphate flame retardants, alkylphenols, pesticide residues, microplastic-associated chemicals [32]. These compounds are continuously released from furniture, electronics, flooring materials, synthetic textiles, mattresses, carpets, paints, and building materials [33]. Several exposure sources remain poorly recognized by clinicians, including:

- Waterproof jackets and outdoor equipment;
- Stain-resistant sofas and carpets;
- Yoga mats and synthetic gym flooring;
- Artificial turf;
- Printer toners and office equipment;
- Electronic devices undergoing thermal degradation;
- Vehicle interiors exposed to sunlight.

Cancer survivors, older adults, and frail patients may be particularly vulnerable because of prolonged indoor exposure and reduced environmental mobility [34] (Table 1).

### Personal-care products and cosmetics and occupational and environmental exposure

Personal-care products represent one of the most consistent sources of chronic low-dose exposure [35]. Parabens, phthalates, benzophenone ultraviolet filters, synthetic musks, triclosan, and related compounds are commonly encountered in moisturizers, deodorants, shampoos, hair sprays, perfumes, cosmetics, nail products, sunscreens anti-aging creams. Repeated daily application creates cumulative dermal exposure that may persist for decades [36]. This route may be particularly relevant in women with breast cancer, individuals receiving endocrine therapies, and long-term cancer survivors, populations in whom hormonal signaling pathways remain clinically important [37]. Occupational exposure remains an underappreciated determinant of EDC burden. Higher exposure levels have been reported among agricultural workers, plastic industry employees, firefighters, textile workers, beauty professionals, healthcare personnel, industrial manufacturing workers [38]. Environmental contamination can additionally occur through air pollution, agricultural runoff, industrial emissions, contaminated soil, and waste disposal sites. Importantly, EDC exposure frequently reflects environmental and socioeconomic inequalities, highlighting the need to consider social determinants of health within future cardio-oncology research frameworks (Table 1).

### Medical and healthcare-related exposure

A largely overlooked issue in cardio-oncology is healthcare-associated exposure. Cancer patients frequently undergo repeated contact with medical materials containing plasticizers and polymer-associated chemicals [39]. Historically, phthalates have been detected in intravenous infusion systems, chemotherapy tubing, blood bags, central venous catheters, enteral feeding devices, dialysis equipment and extracorporeal circulation systems [40]. Although regulatory efforts have reduced the use of some compounds, repeated hospitalization and device-intensive care may contribute to cumulative exposure over time [41]. This concept is particularly intriguing in cardio-oncology because patients simultaneously experience exposure to cardiotoxic therapies and environmental chemicals capable of targeting similar biological pathways, including mitochondrial dysfunction, endothelial injury, inflammation, thrombosis, and metabolic stress (Table 1).

### Microplastics and nanoplastics: emerging endocrine-relevant exposures

An emerging area of considerable interest concerns microplastics and nanoplastics (MNPs), which are increasingly recognized not only as environmental contaminants but also as potential endocrine-disrupting entities [42]. Recent evidence indicates that MNPs are detectable in human blood and multiple tissues, supporting the concept that exposure is systemic rather than confined to the gastrointestinal tract [42]. Beyond their intrinsic biological activity, MNPs may function as carriers of endocrine-disrupting chemicals, including bisphenols, phthalates, PFAS, and other environmental toxicants, thereby acting as vectors that facilitate combined and potentially synergistic exposures [43]. Experimental studies suggest that MNPs can interfere with thyroid, adrenal, ovarian, and pancreatic endocrine function, while simultaneously promoting oxidative stress, inflammatory signaling, mitochondrial dysfunction, and cellular injury [44]. Importantly, MNPs may represent a unique exposomic challenge because they combine physical particle effects with chemical toxicity and endocrine disruption [45]. Although direct evidence in cardio-oncology is currently lacking, the widespread human exposure to MNPs and their ability to interact with pathways implicated in both cancer progression and cardiovascular disease suggest that they should be considered an emerging component of the cardio-oncology exposome requiring further investigation [46]. A clinically relevant concept is that of “pseudo-persistence” [47]. Although several EDCs, including bisphenols, phthalates, parabens and triclosan, are rapidly metabolized and excreted, continuous exposure through food packaging, indoor dust, cosmetics, personal-care products, medical devices and consumer materials may maintain a quasi-constant internal burden [48]. Thus, non-persistent chemicals may generate chronic biological effects through repeated daily exposure, a pattern that is highly relevant to cancer survivors and patients undergoing repeated medical care [49].

## Endocrine disruptors, adipose tissue dysfunction, and cardiovascular vulnerability in cardio-oncology

### Shared biological pathways between endocrine disruptors and cancer therapy–related cardiovascular toxicity

The clinical relevance of EDs in cardio-oncology does not currently derive from direct outcome studies, which remain scarce, but rather from the remarkable overlap between the biological pathways targeted by EDs and those implicated in cancer therapy–related cardiovascular toxicity (CTR-CVT) [50, 51]. Contemporary cardio-oncology recognizes cardiovascular toxicity as a complex and multifactorial process extending far beyond left ventricular dysfunction [52]. Anthracyclines, HER2-targeted therapies, VEGF inhibitors, immune checkpoint inhibitors, radiotherapy, and endocrine therapies can induce a broad spectrum of cardiovascular abnormalities, including myocardial injury, endothelial dysfunction, hypertension, thrombosis, accelerated atherosclerosis, fibrosis, and adverse cardiac remodeling [52–54].

Notably, many of these pathological processes are also consistently linked to chronic exposure to endocrine-disrupting chemicals [55]. Experimental and epidemiological evidence indicates that several EDC classes, including bisphenols, phthalates, PFAS, and persistent organic pollutants, promote oxidative stress, mitochondrial dysfunction, impaired nitric oxide bioavailability, endothelial activation, inflammatory signaling, calcium dysregulation, and epigenetic remodeling [56]. Collectively, these mechanisms may reduce cardiovascular resilience and lower the threshold at which cancer therapies trigger clinically overt cardiovascular toxicity [57]. From a myocardial perspective, mitochondrial dysfunction represents a particularly important point of convergence. Anthracyclines and several targeted therapies induce mitochondrial DNA damage, impaired oxidative phosphorylation, excessive reactive oxygen species generation, and progressive cardiomyocyte injury [58]. Similar mitochondrial abnormalities have been described following exposure to multiple endocrine disruptors, suggesting that chronic environmental exposures may contribute to a pre-existing state of metabolic vulnerability before anticancer therapy is even initiated [59]. The vascular compartment may be equally important.

**Fig. 1:**
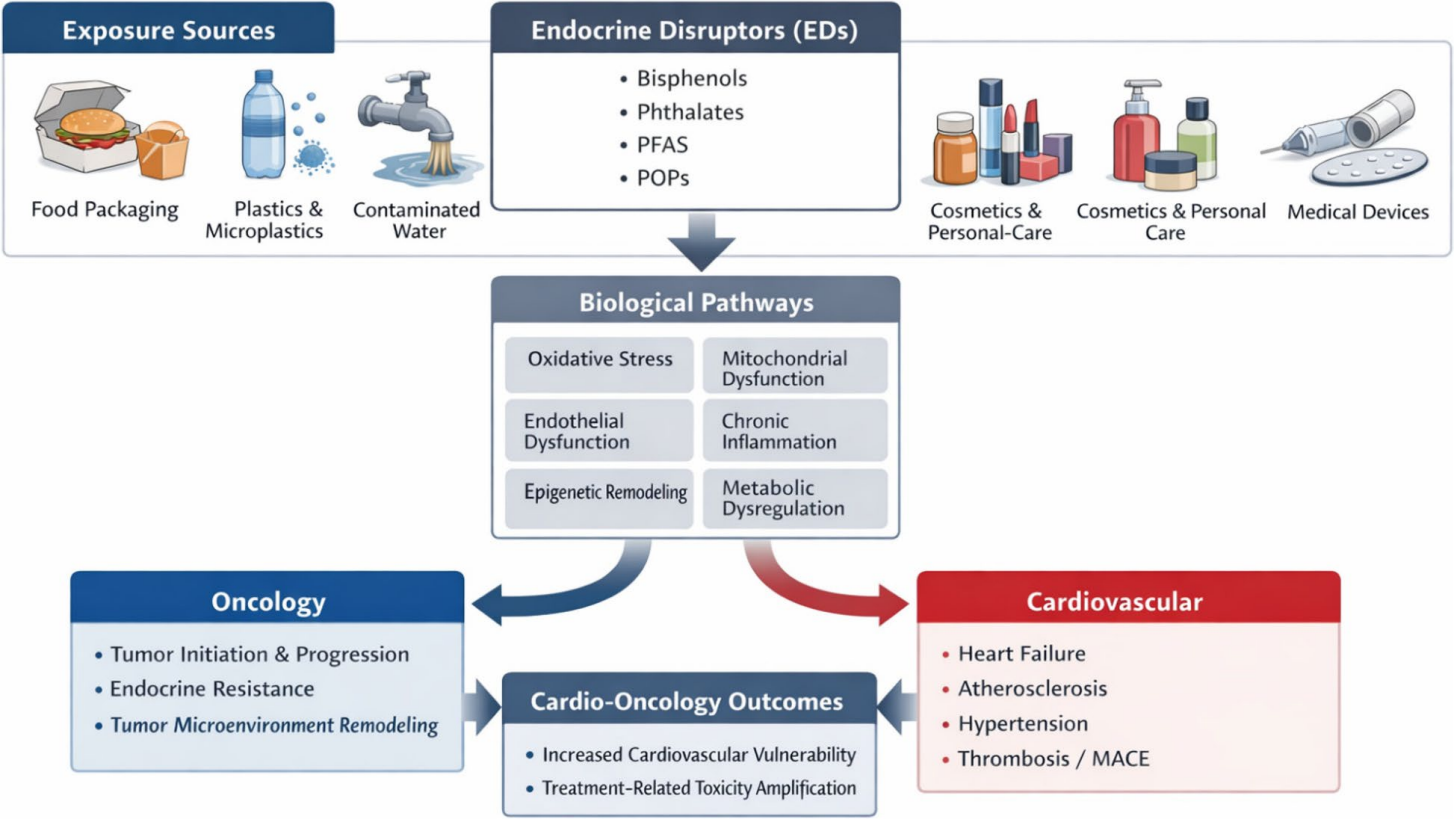
Endocrine disruptors as exposomic modifiers in cardio-oncology. Environmental exposures arising from food pack-aging, plastics, contaminated water, personal-care products, and medical materials lead to chronic contact with endocrine-disrupting chemicals (EDs), including bisphenols, phthalates, per- and polyfluoroalkyl substances (PFAS), and persistent organic pollutants (POPs). These compounds converge on shared biological pathways, namely oxidative stress, mitochondrial dysfunction, endothelial impairment, chronic inflammation, epigenetic remodeling, and metabolic dysregulation. Through these mechanisms, EDs may influence both tumor biology and cardiovascular systems, promoting tumor progression, endocrine resistance, and microenvironmental changes, while simultaneously contributing to heart failure, atherosclerosis, hypertension, and thrombosis. The convergence of these processes may ultimately increase susceptibility to adverse cardio-oncology outcomes and amplify treatment-related cardiovascular toxicity

Endothelial dysfunction is increasingly recognized as a central driver of hypertension, coronary artery disease, thrombosis, and accelerated vascular aging in cancer survivors. Independent of cancer therapy, EDC exposure has been associated with impaired endothelial function, vascular inflammation, reduced nitric oxide signaling, and pro-atherogenic remodeling, raising the possibility that environmental exposures may contribute to interindividual variability in cardiovascular outcomes among patients receiving potentially cardiotoxic treatments [60]. As illustrated in Fig. 1, many of the biological pathways activated by endocrine disruptors converge on the same myocardial and vascular targets involved in CTR-CVT, supporting the concept that EDCs may act as chronic background modifiers of cardiovascular susceptibility rather than isolated toxic agents.

### Epicardial adipose tissue as a potential toxicological niche

An emerging and particularly intriguing hypothesis involves the role of adipose tissue as a biological reservoir for lipophilic endocrine disruptors [61]. Unlike non-persistent compounds that are rapidly metabolized and excreted, several persistent pollutants, including organochlorine compounds, PCBs, dioxins, brominated flame retardants, and selected PFAS, accumulate within adipose tissue and may remain detectable for years or even decades [62]. This concept is highly relevant to cardio-oncology because adipose tissue is now recognized as an active endocrine and immunometabolic organ rather than a passive energy depot. In obesity, metabolic syndrome, diabetes, and cancer survivorship, adipose tissue undergoes profound remodeling characterized by macrophage infiltration, oxidative stress, adipokine imbalance, and chronic low-grade inflammation [63]. Among adipose depots, epicardial adipose tissue (EAT) deserves particular attention. Owing to its direct anatomical continuity with the myocardium and coronary arteries and the absence of a fascial barrier, EAT can exert local paracrine and vasocrine effects on adjacent cardiovascular structures [64]. In pathological states, EAT shifts toward a pro-inflammatory and profibrotic phenotype associated with coronary artery disease, atrial fibrillation, heart failure, and major adverse cardiovascular events [65].

**Fig. 2:**
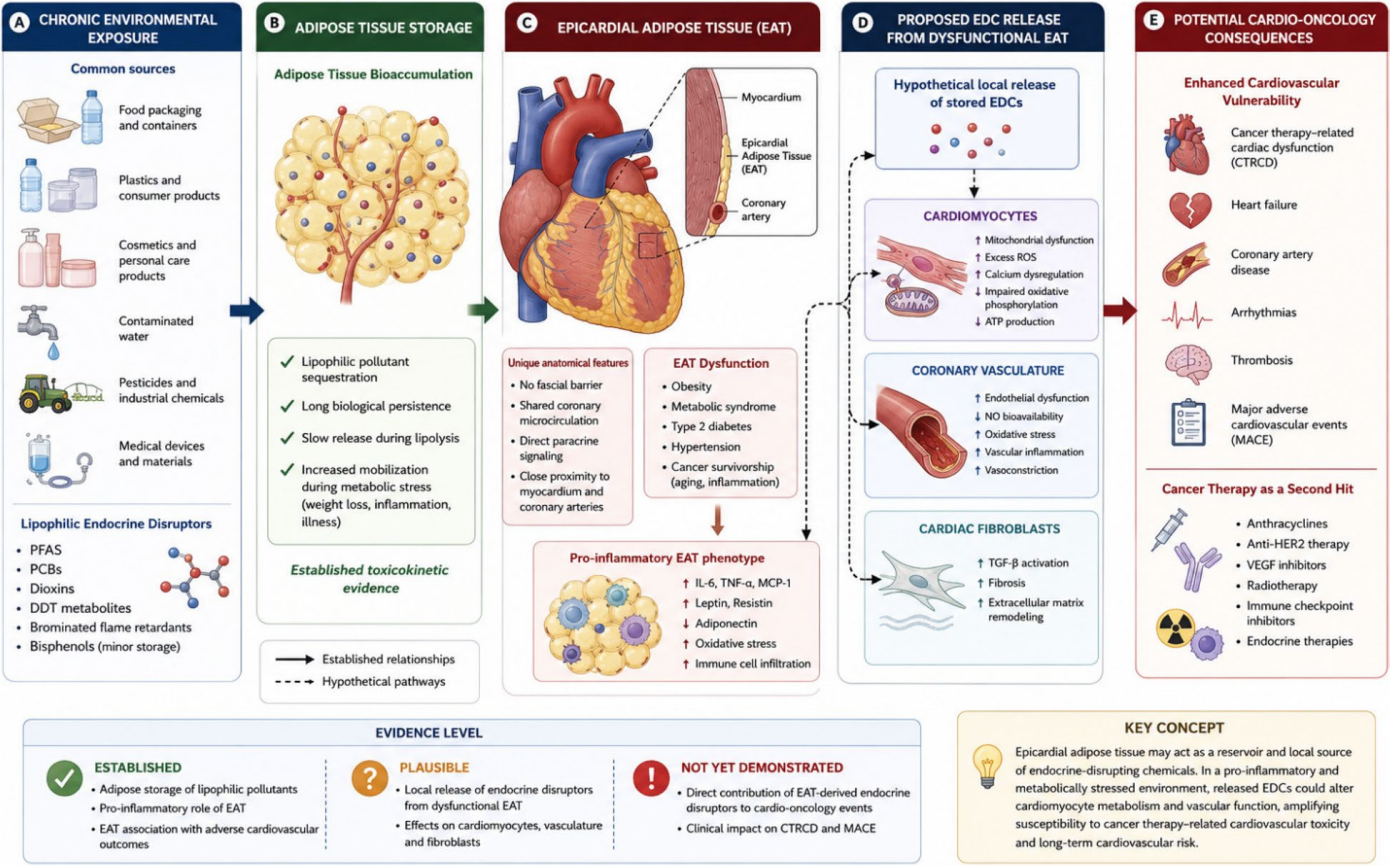
Epicardial adipose tissue as a potential reservoir of endocrine disruptors and a local mediator of cardiovascular vulnerability in cardio-oncology. Lipophilic endocrine disruptors (EDCs), including persistent organic pollutants and selected per- and polyfluoroalkyl substances, can accumulate within adipose depots and remain stored for prolonged periods. Epicardial adipose tissue (EAT), owing to its anatomical contiguity with the myocardium and coronary arteries and its shared microcirculation, may represent a unique interface between environmental exposure and cardiovascular tissues. In conditions such as obesity, metabolic syndrome, diabetes, and cancer survivorship, EAT acquires a pro-inflammatory phenotype characterized by increased cytokine production, adipokine imbalance, and metabolic dysfunction. A biologically plausible but currently unproven hypothesis is that stored EDCs may be released locally from dysfunctional EAT and contribute to myocardial, vascular, and fibrotic remodeling through oxidative stress, mitochondrial dysfunction, endothelial injury, and inflammatory signaling. These mechanisms could potentially amplify susceptibility to cancer therapy–related cardiovascular toxicity and major adverse cardiovascular events. Solid arrows indicate established biological relationships; dashed arrows indicate hypothetical pathways requiring prospective validation

Figure 1 summarizes a biologically plausible model whereby chronic environmental exposure leads to accumulation of lipophilic endocrine disruptors within adipose depots, including EAT. Under conditions of adipose dysfunction, local release of stored pollutants could theoretically contribute to myocardial oxidative stress, endothelial injury, mitochondrial dysfunction, and adverse cardiac remodeling [66]. Although direct evidence supporting this mechanism in humans is currently lacking, each component of the model is individually supported by existing toxicological and cardiovascular literature. At present, endocrine disruptors should not be considered established cardiovascular risk factors in cardio-oncology. However, accumulating evidence supports their role as potential exposomic modifiers capable of interacting with traditional cardiovascular risk factors, metabolic dysfunction, adiposity, and cancer therapies. The greatest clinical relevance may reside in patients with obesity, metabolic syndrome, diabetes, increased epicardial adipose tissue, or pre-existing cardiovascular disease, in whom environmental exposures could amplify biological pathways already activated by cancer therapy. Future studies integrating environmental biomarkers, advanced cardiovascular phenotyping, and adipose tissue biology will be necessary to determine whether endocrine disruptors contribute meaningfully to long-term cardiovascular outcomes in cancer survivors [67]. The cardio-oncology relevance of EDC exposure is strengthened by the growing human evidence linking EDCs to cardiometabolic disease. Contemporary reviews indicate that exposure to several EDC classes is associated with obesity, type 2 diabetes, dyslipidemia, hypertension and cardiovascular disease, with both adult and early-life exposures contributing to later cardiometabolic vulnerability [68]. This is clinically important because these same conditions are major modifiers of cancer therapy-related cardiovascular toxicity and long-term survivorship outcomes (Fig. 2).

## Environmental endocrine disruptors and breast cancer: clinical and molecular implications across disease subtypes

**Fig. 3:**
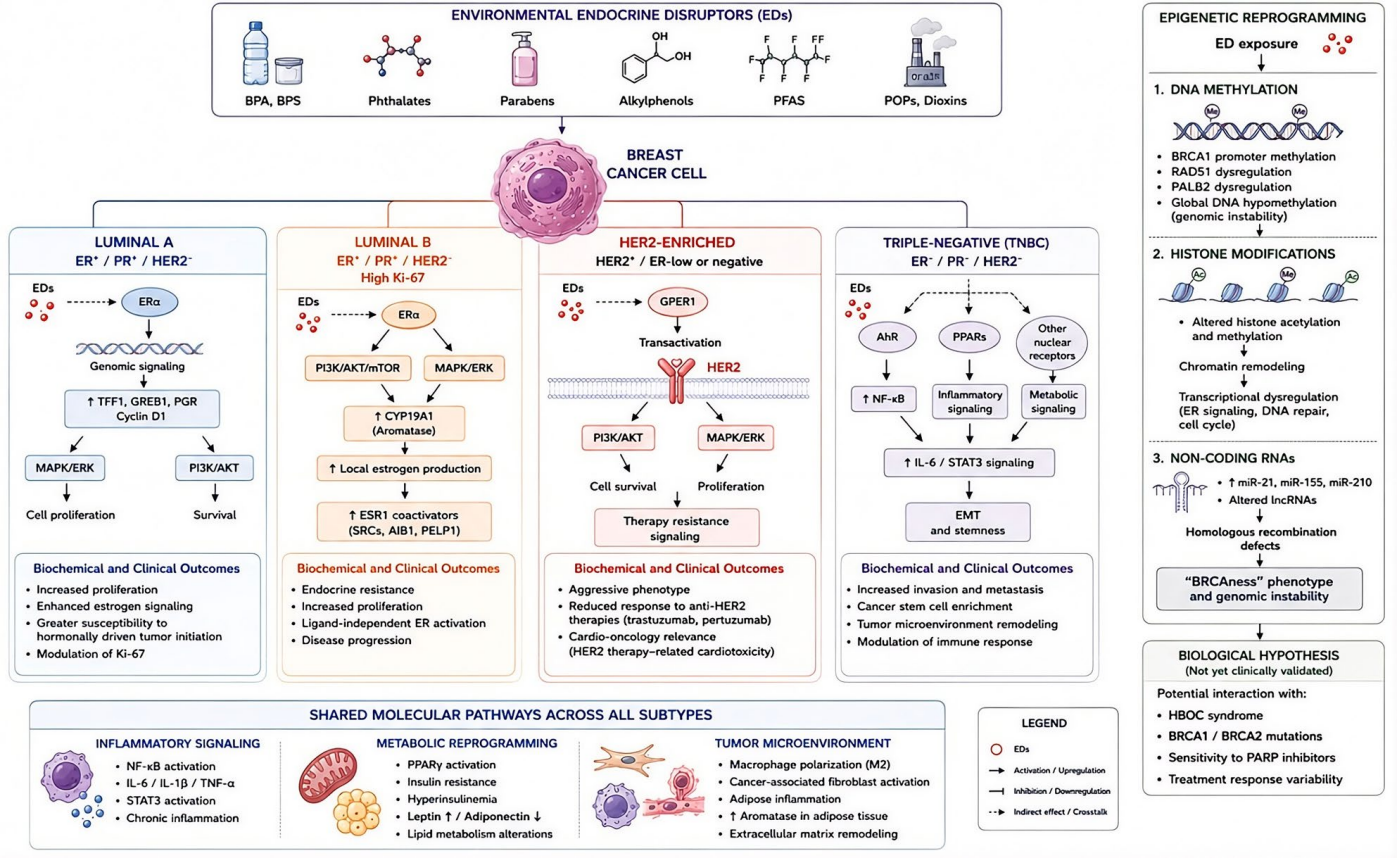
Endocrine disruptors and breast cancer: histotype-specific molecular pathways and epigenetic reprogramming. Environmental endocrine disruptors (EDs), including bisphenols, phthalates, parabens, alkylphenols, per- and polyfluoroalkyl substances (PFAS), and persistent organic pollutants (POPs), may influence breast cancer biology through subtype-specific and shared molecular mechanisms. In luminal tumors, EDs predominantly activate estrogen receptor (ER)-dependent genomic and non-genomic signaling, promoting downstream MAPK/ERK and PI3K/AKT/mTOR pathway activation, enhanced proliferation, and potential endocrine resistance. In HER2-enriched disease, ED-mediated receptor crosstalk involving GPER1 and HER2 may amplify proliferative and survival signaling pathways. In triple-negative breast cancer (TNBC), non-classical pathways, including aryl hydrocarbon receptor (AhR), PPAR signaling, NF-κB activation, and IL-6/STAT3-mediated inflammation, may contribute to tumor progression, epithelial–mesenchymal transition, stemness, and microenvironment remodeling. Across all breast cancer subtypes, ED exposure may converge on common biological processes, including chronic inflammation, metabolic reprogramming, adipose tissue dysfunction, and tumor microenvironment modulation. In parallel, EDs can induce epigenetic alterations through DNA methylation changes, histone remodeling, and dysregulation of non-coding RNAs, potentially affecting homologous recombination pathways and genes involved in hereditary breast and ovarian cancer susceptibility, including BRCA1, BRCA2, PALB2, and RAD51. While several of these mechanisms are supported by experimental and translational evidence, their clinical relevance in human breast cancer progression and therapeutic response requires further prospective investigation

Breast cancer represents the most clinically relevant model through which to examine the oncologic consequences of ED exposure [69]. Unlike many other malignancies, breast cancer development and progression are profoundly influenced by hormonal signaling, nuclear receptor activity, metabolic status, and epigenetic regulation, all biological domains known to be affected by environmental endocrine-active chemicals [70]. Importantly, the effects of EDs are unlikely to be uniform across breast cancer subtypes. As summarized in Fig. 3, susceptibility appears to depend on the underlying molecular architecture of the tumor, with distinct signaling pathways potentially involved in luminal, HER2-enriched, and triple-negative disease.

### Hormone receptor–positive breast cancer: the strongest clinical signal

Among all breast cancer subtypes, the most consistent epidemiologic and biological evidence linking EDs to carcinogenesis concerns hormone receptor–positive disease, particularly in postmenopausal women [71]. Several observational studies and biomonitoring investigations have reported associations between higher exposure to bisphenols, phthalates, selected organochlorine compounds, and increased breast cancer risk, although results remain heterogeneous across populations and exposure assessment methods. The biological rationale is compelling. Following menopause, circulating ovarian estrogen production declines substantially, increasing the relative importance of peripheral estrogen synthesis within adipose tissue and potentially enhancing susceptibility to weak exogenous estrogenic signals [72]. Under these conditions, chronic low-dose exposure to compounds such as BPA, BPS, parabens, alkylphenols, and selected phthalates may exert measurable biological effects despite their substantially lower receptor affinity compared with endogenous estrogens [73]. As illustrated in Fig. 3, EDs can activate both genomic and non-genomic estrogen receptor pathways, promoting downstream PI3K/AKT/mTOR and MAPK/ERK signaling, two molecular axes strongly implicated in breast cancer proliferation, survival, and treatment resistance [74]. Experimental evidence further suggests that some EDs may increase aromatase (CYP19A1) expression within adipose tissue, enhancing local estrogen production and reinforcing a pro-tumorigenic microenvironment [75]. Particular attention should be given to Luminal B tumors, where greater proliferative activity and signaling redundancy may increase vulnerability to environmental modulation. In this setting, ED exposure has been proposed as a potential contributor to endocrine resistance through ligand-independent estrogen receptor activation, coactivator recruitment, and persistent activation of growth factor signaling pathways. Although direct clinical evidence linking ED burden to endocrine therapy failure remains limited, this hypothesis is increasingly supported by mechanistic and translational studies [76]. Taken together, current evidence suggests that endocrine disruptors are unlikely to function as major independent carcinogens in hormone receptor–positive breast cancer. Rather, they may act as long-term biological modifiers capable of amplifying estrogenic signaling, influencing tumor behavior, and potentially contributing to interindividual variability in disease progression and therapeutic response, particularly among postmenopausal women with obesity, metabolic dysfunction, or increased adipose tissue burden [77]. Importantly, epidemiological observations provide partial support for this biological framework. While early case–control studies evaluating urinary bisphenol A concentrations in postmenopausal women failed to demonstrate a consistent association with breast cancer risk, larger biomonitoring and prospective investigations have produced mixed results [78]. In the Women's Health Initiative, urinary phthalate metabolites were not significantly associated with incident postmenopausal breast cancer, highlighting the complexity of exposure assessment in diseases characterized by long latency periods. Conversely, studies measuring circulating BPA concentrations have reported positive associations with incident breast cancer, suggesting that systemic bioactive exposure may be more relevant than short-term urinary biomarkers [79]. Importantly, recent systematic reviews integrating epidemiological evidence concluded that several endocrine-disrupting chemicals, particularly persistent compounds with estrogenic properties, may contribute to breast cancer risk, although substantial heterogeneity and residual confounding remain. Collectively, these findings suggest that endocrine disruptors are unlikely to function as dominant carcinogens, but may act as long-term environmental modifiers capable of amplifying hormonal and metabolic pathways involved in breast cancer development, particularly in postmenopausal women.

### HER2-enriched disease: receptor crosstalk and signaling amplification

HER2-positive tumors provide a second biologically plausible setting for ED-mediated effects [80]. Experimental studies suggest that several endocrine disruptors can activate membrane-associated estrogen signaling pathways, particularly through G protein-coupled estrogen receptor 1 (GPER1), resulting in downstream transactivation of HER2-related signaling networks [81]. This interaction may enhance PI3K/AKT and MAPK/ERK activation, pathways already central to HER2-driven tumor biology. Although direct clinical evidence remains limited, these observations raise the possibility that environmental exposures may contribute to interindividual variability in tumor behavior and therapeutic response [82]. From a cardio-oncology perspective, this interaction is particularly intriguing because HER2 signaling is relevant not only to tumor growth but also to myocardial homeostasis, providing a potential biological intersection between cancer progression and treatment-related cardiotoxicity [83].

### Triple-negative breast cancer: beyond estrogen signaling

The relationship between EDs exposure and triple-negative breast cancer (TNBC) appears to be less dependent on classical hormonal pathways and more closely linked to inflammation, metabolic signaling, and microenvironmental remodeling [84]. As illustrated in Fig. 3, activation of aryl hydrocarbon receptor (AhR), PPAR-related pathways, NF-κB signaling, and IL-6/STAT3 inflammatory networks may contribute to epithelial–mesenchymal transition, cancer stem-cell maintenance, immune modulation, and metastatic dissemination [85]. Although current evidence does not support EDs as primary drivers of TNBC, they may act as disease modifiers capable of influencing tumor aggressiveness and progression.

### Shared biological and epigenetic mechanisms

Despite important subtype-specific differences, several biological processes appear to be consistently affected by endocrine disruptor exposure across breast cancer phenotypes (Fig. 3). These include chronic inflammatory activation, metabolic reprogramming, adipose tissue dysfunction, local aromatase induction, insulin resistance, and remodeling of the tumor microenvironment [86]. Notably, many of these mechanisms overlap with pathways implicated in cardiovascular disease and cancer therapy–related cardiovascular toxicity, reinforcing the broader cardio-oncology relevance of environmental exposures [87]. A particularly important emerging area is epigenetic reprogramming. Experimental studies demonstrate that EDs can alter DNA methylation, histone modification patterns, and non-coding RNA expression, thereby generating persistent changes in gene regulation long after exposure has occurred [87]. Of particular interest are reports linking ED exposure to altered regulation of homologous recombination pathways and genes involved in hereditary breast and ovarian cancer susceptibility, including BRCA1, BRCA2, PALB2, and RAD51 [88]. While current evidence does not support a direct effect of ED exposure on BRCA mutation penetrance, these observations suggest that environmental exposures may contribute to a broader "*BRCAness-like*" phenotype characterized by genomic instability and altered DNA repair capacity [89]. Taken together, current evidence supports the concept that endocrine disruptors act less as classical carcinogens and more as long-term modifiers of breast cancer biology. Their effects appear to be mediated through a combination of receptor signaling, metabolic dysregulation, inflammatory activation, tumor microenvironment remodeling, and epigenetic reprogramming (Fig. 3). Although substantial gaps remain, particularly regarding causal human data, this emerging exposomic framework provides a biologically coherent model linking environmental exposure, host metabolism, tumor behavior, and cardiovascular vulnerability [90]. Future prospective studies integrating exposure biomarkers, molecular tumor profiling, and clinical outcomes will be necessary to determine whether endocrine disruptor burden contributes meaningfully to breast cancer progression, treatment response, and survivorship [91]. A further dimension is developmental susceptibility. Prenatal, early-life and pubertal exposure to EDCs may alter endocrine, metabolic and cardiovascular programming, increasing later susceptibility to obesity, insulin resistance, dyslipidemia and vascular dysfunction. This concept may be relevant to young cancer survivors, in whom environmental exposures, early metabolic programming and cardiotoxic cancer therapies may converge over decades to shape long-term cardiovascular risk [92].

## Environmental endocrine disruptors as emerging determinants of cardiovascular vulnerability

**Fig. 4:**
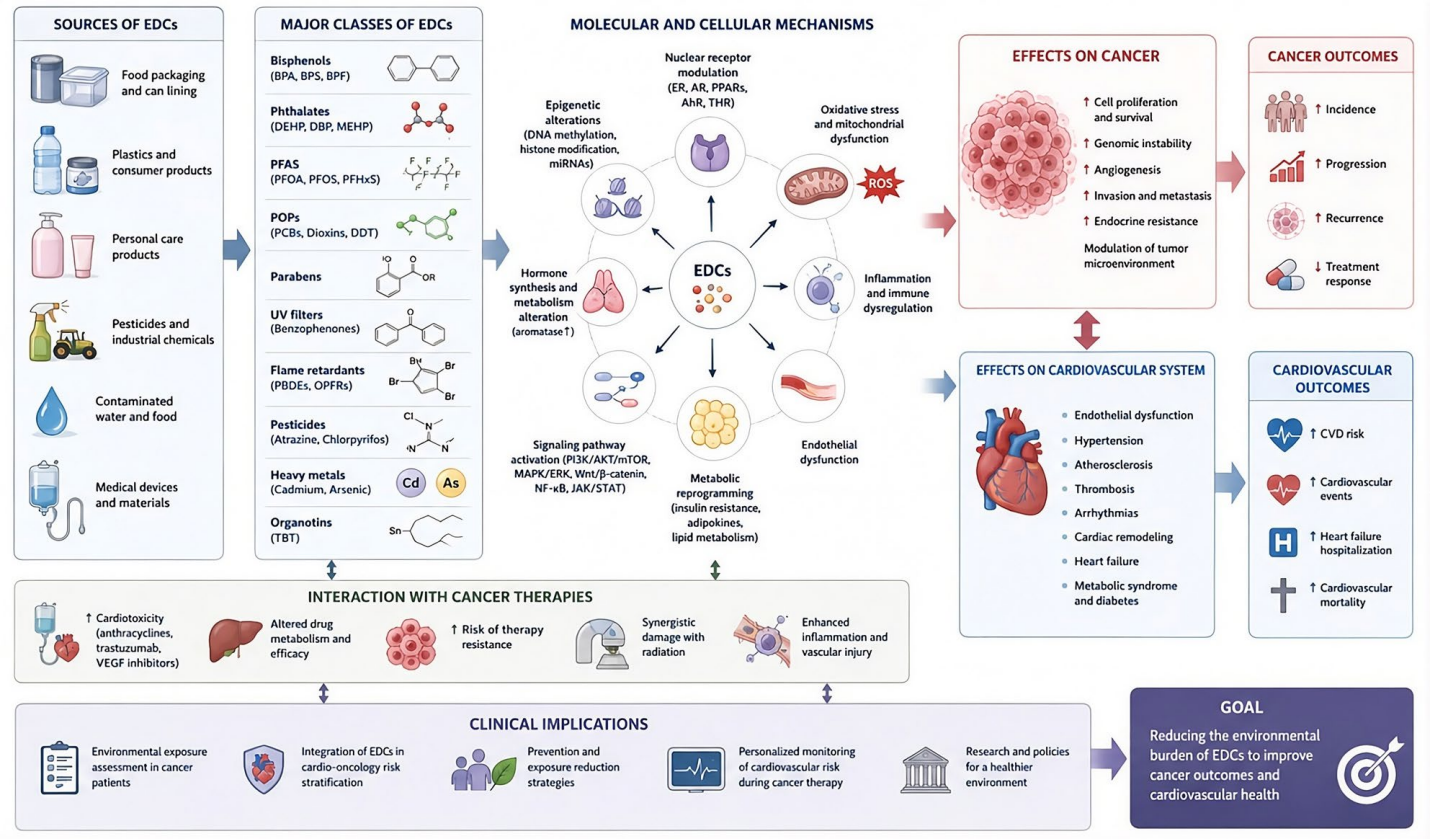
Endocrine-disrupting chemicals (EDCs) as exposomic modifiers in cardio-oncology. Chronic environmental exposure to endocrine-disrupting chemicals (EDCs) occurs through food packaging, plastics, contaminated water, cosmetics, pesticides, industrial pollutants, and medical materials. Major EDC classes, including bisphenols, phthalates, per- and polyfluoroalkyl substances (PFAS), persistent organic pollutants (POPs), parabens, flame retardants, pesticides, and heavy metals, interact with multiple molecular and cellular pathways relevant to both cancer biology and cardiovascular disease. EDCs modulate nuclear receptor signaling, oxidative stress, mitochondrial function, endothelial integrity, inflammatory pathways, metabolic homeostasis, and epigenetic regulation, thereby influencing tumor progression, endocrine resistance, vascular dysfunction, thrombosis, and myocardial injury. Within the cardio-oncology setting, these mechanisms may amplify susceptibility to cancer therapy–related cardiovascular toxicity and adverse long-term cardiovascular outcomes. The figure summarizes the proposed role of EDCs as cross-cutting exposomic contributors linking oncologic and cardiovascular vulnerability

The cardiovascular consequences of EDs exposure have emerged as an increasingly important area of investigation over the past decade. Although individual compounds differ substantially in their biological activity, a consistent body of epidemiological, translational, and experimental evidence suggests that chronic exposure to endocrine-disrupting chemicals may contribute to cardiovascular disease through a limited number of interconnected mechanisms, including endothelial dysfunction, metabolic dysregulation, oxidative stress, inflammation, mitochondrial injury, and thrombosis [93]. These pathways are highly relevant to contemporary cardio-oncology because they substantially overlap with those implicated in cancer therapy-related cardiovascular toxicity. As illustrated in Fig. 4, environmental exposure to EDs originates from multiple sources encountered throughout daily life, including food packaging, plastics, personal care products, contaminated water, pesticides, industrial pollutants, and healthcare-related materials. Following exposure, several classes of EDs,including bisphenols, phthalates, PFAS, persistent organic pollutants, parabens, flame retardants, pesticides, and selected heavy metals, can interact with hormonal, metabolic, inflammatory, and epigenetic pathways, ultimately affecting both cardiovascular and cancer-related biological processes [94].

### Endothelial dysfunction and accelerated vascular aging

Among the various mechanisms proposed, endothelial dysfunction appears to represent the most consistent and clinically relevant pathway linking ED exposure to cardiovascular disease. Experimental studies have shown that multiple ED classes impair nitric oxide bioavailability, increase oxidative stress, activate inflammatory signaling, and promote endothelial activation [95]. These alterations favor vascular stiffness, impaired vasodilatory responses, leukocyte recruitment, and pro-atherogenic remodeling. This biological framework is supported by human studies reporting associations between ED exposure and hypertension, coronary artery disease, and adverse cardiovascular health metrics [56]. From a clinical perspective, endothelial dysfunction is particularly relevant because it represents a common upstream mechanism for several cardiovascular manifestations frequently encountered in cancer survivors, including accelerated atherosclerosis, coronary artery disease, thrombotic complications, and vascular aging.

### Cardiometabolic dysfunction, heart failure, and major cardiovascular events

A second major pathway involves cardiometabolic dysregulation. Increasing evidence links chronic ED exposure to obesity, insulin resistance, dyslipidemia, metabolic syndrome, and adipose tissue dysfunction [96]. These associations are especially relevant in cardio-oncology, where metabolic abnormalities frequently coexist with cancer treatment-related cardiovascular stress. Several ED classes have been associated with alterations in mitochondrial function, impaired cellular energetics, calcium handling abnormalities, and chronic low-grade inflammation [97]. Collectively, these mechanisms may contribute to myocardial remodeling and progressive loss of cardiovascular reserve. Although direct clinical evidence linking ED exposure to incident heart failure remains limited, observational studies have reported associations between selected bisphenol analogues, phthalate metabolites, and markers of subclinical cardiac dysfunction [55]. More importantly, the consistent relationship between ED exposure and cardiometabolic disease strongly supports an indirect contribution to heart failure development, particularly in obesity-related heart failure and heart failure with preserved ejection fraction. The same biological processes likely contribute to the increased incidence of major adverse cardiovascular events (MACE) observed in several population-based studies [98]. While EDs cannot yet be considered independent predictors of cardiovascular events, cumulative environmental exposure appears capable of reducing cardiovascular resilience and amplifying the impact of traditional risk factors, including obesity, diabetes, chronic kidney disease, and hypertension.

### Thrombosis and procoagulant signaling

The relationship between EDs exposure and thrombotic disease remains less extensively characterized but is biologically plausible [99]. Several endocrine-active compounds possess estrogenic or pseudo-estrogenic properties capable of influencing platelet activation, endothelial injury, coagulation signaling, and fibrinolytic balance. Experimental studies have demonstrated increased platelet reactivity following exposure to selected plastic-derived chemicals, including bisphenol A [100]. Although definitive human evidence linking EDs burden to venous thromboembolism or arterial thrombosis remains limited, current data support the concept that chronic exposure may contribute to a prothrombotic vascular phenotype characterized by endothelial activation, inflammation, and altered platelet function [101]. The major implication for cardio-oncology is that endocrine disruptors may influence the same biological pathways targeted by cancer therapies (Fig. 4). Oxidative stress, endothelial dysfunction, inflammation, metabolic reprogramming, mitochondrial injury, and epigenetic remodeling are now recognized hallmarks of both environmental toxicology and cancer therapy-related cardiovascular toxicity. Consequently, EDs should not currently be viewed as established cardiovascular risk factors comparable to hypertension or diabetes. Rather, they may represent chronic exposomic modifiers capable of influencing individual susceptibility to cardiovascular injury. This concept may be particularly relevant in patients with obesity, metabolic syndrome, increased visceral or epicardial adiposity, pre-existing cardiovascular disease, or exposure to cardiotoxic therapies such as anthracyclines, HER2-targeted agents, VEGF inhibitors, thoracic radiotherapy, and endocrine therapies [102, 103].

Taken together, the available evidence supports a conceptual model in which endocrine disruptors contribute to a background state of cardiovascular vulnerability that interacts with host metabolic characteristics and cancer therapies to influence long-term cardiovascular outcomes. As summarized in Fig. 4, this integrated exposomic framework provides a biologically plausible bridge between environmental health, cardiovascular disease, and modern cardio-oncology.

## Reducing endocrine disruptor exposure in cancer survivors: practical implications for cardiovascular prevention

Although direct intervention trials evaluating EDs reduction in cardio-oncology are currently lacking, growing evidence suggests that environmental exposure may represent a modifiable component of long-term cardiovascular risk [104]. Because most endocrine-disrupting chemicals enter the human body through everyday interactions with food systems, consumer products, water, and indoor environments, exposure reduction can often be achieved through relatively simple behavioral modifications. Importantly, these interventions overlap substantially with established recommendations for cardiovascular prevention and healthy cancer survivorship, making them particularly attractive in clinical practice [105].

### Food systems as the major modifiable exposure source

For most individuals, diet represents the principal route of exposure to bisphenols, phthalates, PFAS, and numerous packaging-associated chemicals [106]. Consequently, the most effective exposure-reduction strategies focus not only on food selection but also on food processing, storage, and preparation. Patients should be encouraged to preferentially consume fresh or minimally processed foods while limiting frequent consumption of canned products, ultra-processed foods, ready-to-eat meals, and takeaway products packaged in plastic-containing materials. Common but often overlooked sources include canned soups, canned legumes, canned tuna, canned soft drinks, microwave-ready meals, plastic-wrapped cheeses, pre-packaged salads, and hot takeaway foods served in plastic containers. Replacing these products with fresh, frozen, or glass-packaged alternatives may substantially reduce exposure to bisphenols and phthalates. Particular attention should be paid to food heating practices [107]. Heating food in plastic containers, covering hot meals with plastic films, microwaving leftovers in plastic packaging, or storing hot beverages in plastic vessels may increase migration of endocrine-active compounds into food. For this reason, glass, ceramic, porcelain, and stainless-steel containers should be preferred whenever possible.

### Dietary patterns and internal chemical burden

The composition of the diet itself may also influence cumulative exposure. Several persistent endocrine disruptors accumulate along the food chain and may reach higher concentrations in animal fat. Consequently, dietary patterns emphasizing vegetables, fruits, legumes, whole grains, nuts, and fish from lower-contaminant sources may reduce exposure while simultaneously improving cardiovascular health [108]. This observation is particularly relevant in cardio-oncology, where Mediterranean dietary patterns are already recommended because of their beneficial effects on inflammation, endothelial function, insulin sensitivity, and body composition. Notably, the same dietary pattern may simultaneously reduce exposure to several endocrine-disrupting chemicals, creating a potential dual benefit for cancer survivors. Practical measures include thorough washing of fruits and vegetables, reducing consumption of heavily processed meats, trimming visible animal fat when appropriate, and following regional recommendations regarding fish species with lower contaminant burdens [109].

### Water, personal-care products, and indoor environments

Exposure reduction extends beyond nutrition. Drinking water may represent a relevant source of PFAS and other contaminants in certain geographical areas [110]. When local contamination is known or suspected, the use of appropriate filtration systems and storage of beverages in glass or stainless-steel containers may be considered. Personal-care products represent another important and often underestimated source of exposure. Fragrances, perfumes, deodorants, shampoos, cosmetics, moisturizers, nail products, and sunscreens may contain phthalates, parabens, benzophenones, and other endocrine-active compounds [111]. While complete avoidance is neither realistic nor necessary, choosing fragrance-free products and limiting unnecessary use of highly scented formulations may reduce cumulative exposure. Similarly, the indoor environment contributes substantially to chronic exposure. Household dust frequently contains phthalates, flame retardants, PFAS, and plastic-associated chemicals released from furniture, electronics, flooring materials, and textiles. Regular ventilation, wet cleaning, hand hygiene before meals, removal of shoes indoors, and reduction of unnecessary soft-vinyl products represent practical measures that can be incorporated into everyday life without major lifestyle disruption. From a clinical perspective, endocrine disruptor reduction should not be viewed as an alternative or complementary medicine strategy, but rather as an extension of contemporary preventive cardio-oncology. The same patients most vulnerable to cancer therapy-related cardiovascular toxicity, those with obesity, metabolic syndrome, diabetes, hypertension, increased visceral or epicardial adiposity, or pre-existing cardiovascular disease, are also likely to be those most susceptible to the biological effects of chronic environmental exposures. Accordingly, clinicians may consider incorporating a brief environmental exposure assessment into survivorship care, particularly among high-risk patients [112]. Counseling focused on food packaging, plastic use, water quality, household exposures, and personal-care products can be delivered alongside traditional recommendations regarding diet, physical activity, smoking cessation, and weight management. At present, there is no evidence supporting detoxification protocols, supplements, or commercial cleansing programs. Instead, the most scientifically grounded approach remains the progressive reduction of cumulative daily exposure through sustainable lifestyle modifications. In this regard, endocrine disruptor reduction may represent a novel but immediately actionable component of precision survivorship and cardiovascular prevention [113].

## Conclusion

The rapid expansion of the cancer survivor population has transformed cardiovascular disease into a major determinant of long-term outcomes in oncology. Although contemporary cardio-oncology has focused primarily on treatment-related toxicity, increasing evidence suggests that environmental exposures may represent an additional and largely overlooked layer of cardiovascular risk. Endocrine disruptors are unlikely to act as isolated causal agents. Rather, they should be viewed as chronic exposomic modifiers capable of interacting with host susceptibility, metabolic dysfunction, adipose tissue biology, and cancer therapies. The biological convergence between endocrine disruptor toxicity and the mechanisms underlying cancer therapy-related cardiovascular toxicity, including oxidative stress, mitochondrial dysfunction, endothelial injury, inflammation, thrombosis, metabolic remodeling, and epigenetic reprogramming, is striking and clinically relevant. From a clinical perspective, the key message is not that endocrine disruptors have already been proven to cause cardiovascular toxicity in patients with cancer, but that they may contribute to interindividual variability in cardiovascular outcomes and survivorship trajectories. This concept is particularly relevant in patients with obesity, diabetes, metabolic syndrome, increased visceral or epicardial adiposity, pre-existing cardiovascular disease, or exposure to highly cardiotoxic treatments, where environmental exposures may act as a biologically meaningful second hit. Importantly, environmental exposure differs from many traditional cardiovascular risk modifiers because it is potentially actionable. While definitive intervention trials remain unavailable, exposure-reduction strategies are largely consistent with established recommendations for cardiovascular prevention and healthy cancer survivorship. Consequently, they represent low-risk interventions that can reasonably be incorporated into comprehensive cardio-oncology care. Future cardio-oncology research should move beyond a therapy-centered paradigm and incorporate the exposome into cardiovascular risk assessment. Prospective studies integrating standardized EDC biomonitoring, repeated exposure measurements, adipose tissue phenotyping, advanced imaging, molecular profiling, cardiometabolic phenotyping, and adjudicated cardiovascular outcomes are needed to determine whether endocrine disruptors contribute meaningfully to treatment-related cardiovascular toxicity and long-term survivorship. The next frontier of cardio-oncology may not be confined to the cancer cell or the myocardium, but may also involve the environmental exposures that continuously shape both. Understanding and mitigating endocrine disruptor burden could therefore represent an important missing component of precision prevention in cancer survivors.

## Data Availability

No datasets were generated or analysed during the current study.

## Abbreviations

AhR: Aryl Hydrocarbon Receptor
AKT: Protein Kinase B
BPA: Bisphenol A
BPAF: Bisphenol AF
BPF: Bisphenol F
BPS: Bisphenol S
BRCA1: Breast Cancer Gene 1
BRCA2: Breast Cancer Gene 2
CTR-CVT: Cancer Therapy-Related Cardiovascular Toxicity
CVD: Cardiovascular Disease
DBP: Dibutyl Phthalate
DEHP: Di(2-ethylhexyl) Phthalate
DINCH: Di(isononyl) Cyclohexane-1,2-dicarboxylate
DINP: Diisononyl Phthalate
DIDP: Diisodecyl Phthalate
DNA: Deoxyribonucleic Acid
DDE: Dichlorodiphenyldichloroethylene
DDT: Dichlorodiphenyltrichloroethane
EAT: Epicardial Adipose Tissue
EDs: Endocrine Disruptors
EDCs: Endocrine-Disrupting Chemicals
ER: Estrogen Receptor
GPER1: G Protein-Coupled Estrogen Receptor 1
HCB: Hexachlorobenzene
HER2: Human Epidermal Growth Factor Receptor 2
IL-6: Interleukin-6
MACE: Major Adverse Cardiovascular Events
MAPK: Mitogen-Activated Protein Kinase
MNPs: Microplastics and Nanoplastics
mTOR: Mammalian Target of Rapamycin
NF-κB: Nuclear Factor Kappa B
PALB2: Partner and Localizer of BRCA2
PBDEs: Polybrominated Diphenyl Ethers
PCBs: Polychlorinated Biphenyls
PCSK9: Proprotein Convertase Subtilisin/Kexin Type 9
PFAS: Per- and Polyfluoroalkyl Substances
PFHxS: Perfluorohexane Sulfonate
PFNA: Perfluorononanoic Acid
PFOA: Perfluorooctanoic Acid
PFOS: Perfluorooctane Sulfonate
PI3K: Phosphoinositide 3-Kinase
POPs: Persistent Organic Pollutants
PPARγ: Peroxisome Proliferator-Activated Receptor Gamma
RAD51: RAD51 Recombinase
ROS: Reactive Oxygen Species
STAT3: Signal Transducer and Activator of Transcription 3
TBT: Tributyltin
TDCIPP: Tris(1,3-dichloro-2-propyl) Phosphate
TNBC: Triple-Negative Breast Cancer
TPHP: Triphenyl Phosphate
VEGF: Vascular Endothelial Growth Factor
